# Compliance to Prenatal Iron and Folic Acid Supplement Use in Relation to Low Birth Weight in Lilongwe, Malawi

**DOI:** 10.3390/nu10091275

**Published:** 2018-09-10

**Authors:** Aaron Thokozani Chikakuda, Dayeon Shin, Sarah S. Comstock, SuJin Song, Won O. Song

**Affiliations:** 1 Department of Food Science and Human Nutrition, Michigan State University, East Lansing, MI 48824, USA; achikakuda@luanar.ac.mw (A.T.C.); comsto37@msu.edu (S.S.C.); 2 Department of Public Health, Food Studies and Nutrition, Syracuse University, Syracuse, NY 13244, USA; dshin03@syr.edu; 3 Department of Food and Nutrition, Hannam University, Daejeon 34054, Korea; sjsong@hnu.kr

**Keywords:** prenatal iron and folic acid (IFA) supplements, low birth weight, maternal anemia, Malawi

## Abstract

Prenatal iron and folic acid (IFA) supplements are offered free to all pregnant women in Malawi to reduce maternal anemia and improve birth outcomes. We investigated the association between self-reported compliance to IFA intake and risk of low birth weight (LBW). Pregnant women who attended Bwaila Maternity Wing of Lilongwe District Hospital for delivery were recruited (*n* = 220). We used a questionnaire to collect self-reported information on IFA use and maternal sociodemographic data. Before delivery, blood samples for maternal hemoglobin (Hb) and folate status, and upon delivery, birth weight, and other newborn anthropometrics were measured. We used multivariable logistic regression to determine risk of LBW by prenatal IFA intake. The self-reported number of IFA pills taken during pregnancy was positively associated with Hb, but not serum and RBC folate concentration: <45, 45–89 and ≥90 pills taken corresponded with mean (SD) Hb 10.7 (1.6), 11.3 (1.8), and 11.7 (1.6) g/dL, respectively (*p* = 0.006). The prevalence of LBW was 20.1%, 13.5% and 5.6% for those who reported taking IFA pills <45, 45–89, and ≥90 pills, respectively (*p* = 0.027). Taking >60 IFA pills reduced risk of LBW delivery (OR (95% CI) = 0.15 (0.03–0.70), *p* = 0.033) than taking ≤30 pills. Self-reported compliance to IFA use is valid for assessing prenatal supplement program in Malawi, especially Hb status, and can reduce the rate of LBW.

## 1. Introduction

Low birth weight (LBW) is internationally recognized as a birth weight below 2500 g (5.5 pounds). LBW is a birth outcome of importance to public health, associated with increased morbidity and mortality in neonates and infants and cardiovascular disease risks later in life [1,2,3]. This practical cutoff for international comparison is based on epidemiological observations that LBW babies are 20 times more likely to die than heavier infants [4].

Over 20 million infants worldwide are born with LBW. More than half of LBW babies are born in developing countries, particularly South-Central Asia, where more than 27% of the babies born weigh less than 2500 g. LBW prevalence in Sub-Saharan Africa (15%) is similar to the level in the Caribbean region (14%). The Central America and Oceania region has a LBW rate of about 10% [4]. LBW is still a leading cause of neonatal and infant mortality in the U.S. and other industrialized countries, although this prevalence is still much lower than that in developing countries [5].

More than half of LBW babies are born in developing countries and Malawi is as affected as other developing countries, with a LBW prevalence of above-the-world average. The national prevalence of LBW in Malawi is 12%, with the highest prevalence seen among mothers younger than 20 years of age (16%) and those older than 35 years of age. The Central region has a higher prevalence of LBW than Northern and Southern regions within Malawi [6].

Micronutrient deficiencies during pregnancy, particularly iron and folate, contribute significantly to the prevalence of LBW [7]. In a recently reported study in China, correction of micronutrient deficiency in pregnancy with prenatal supplementation improved birth outcomes [8]. LBW contributes significantly to neonatal and infant morbidity and mortality in Malawi. Despite the Malawian government’s efforts to improve birth outcomes and health status for Malawians, the prevalence of most health risk indicators remains high. Anemia prevalence has gone up in reproductive age and pregnant women from 28 to 33% and 38 to 45%, from 2010 to 2016, respectively. The current maternal mortality rate of 497/100,000 live births indicates the need to improve maternal and child health. Maternal and infant mortality indicates the level of quality of healthcare available to citizens in a country [9]. For example, a high maternal mortality rate might be from high anemia that negatively affects birth outcomes and health of newborns [10,11]. This might indicate that there are inadequate screening and treatment services available for pregnant women, as evidenced by the high anemia prevalent in reproductive age women and pregnant women in Malawi. The nutritional status of children is poor, as indicated by 37% of children under the age of five being stunted [6].

Micronutrient supplements improve maternal nutrition status and birth outcomes [12,13,14,15]. The Generation R study in Rotterdam, the Netherlands, found that folic acid supplements increased weight at birth by 68 g and placental weight by 13 g in the supplemented group higher than those not supplemented [16]. In another study in northern China, multiple micronutrient supplements, which included IFA, increased birth weight, especially in those who had a low hemoglobin status at baseline [8]. Anemia is one of the biggest contributors of low birth [7,17]. In a case control study in Sudan, antenatal hemoglobin status was found to have modulating effects on birth outcomes, particularly LBW [7].

There have been clinical studies showing the efficacy of supplements in increasing biomarker status. However, these were conducted in controlled environments, while in real life people take medications freely at home. There have been no studies in Malawi showing how self-reported use of prenatal supplements increases hemoglobin and folate status. This in turn will validate self-reported intake of prenatal supplements as a reliable compliance monitoring method, which can be used to predict birth outcomes. We thus hypothesized that the self-reported number of IFA taken during pregnancy is inversely associated with LBW risks mediated by increased levels of maternal hemoglobin.

## 2. Materials and Methods

### 2.1. Study Subjects

The retrospective cross-sectional study design was not pre-specified and was considered exploratory. All pregnant women reporting for delivery at the Bwaila Maternity Wing of Lilongwe District Hospital were asked to participate in the study. Recruitment of subjects followed convenience sampling and included women of all age groups with viable singleton pregnancy or twin gestation delivering at 28 weeks or more. The women resided in Lilongwe district (rural, peri-urban or urban), which is the catchment area for this hospital. We recruited women with pregnancies of various gestation ages (28 weeks and upwards).

The study excluded dyads where the mother had severe anemia (requiring a blood transfusion), the presence of placenta previa (or history of bleeding during pregnancy due to early partial separation of placenta), delivery involved instrumentation, and those where an infant had brain trauma. Additionally, participants must not have had a severe medical condition known to severely affect maternal nutrition status, placenta health, or newborn health. In summary, we excluded all obstetrical and medical emergencies. Babies and mothers that were in intensive care unit (ICU), high dependence unit (HDU), or required constant medical support and monitoring were excluded, so that their medical care was not interrupted. Of the 220 pregnant women who consented to participating in the study, seven who delivered twins were excluded from the final analysis. The final analytic sample size included 213 pregnant women.

### 2.2. Ethical Clearance/Institutional Review Board Approval

Ethical clearance or institutional review board approval was obtained from Michigan State University and the National Health Sciences Research Committee (NHSRC) in Malawi. At the hospital level, the Lilongwe District Health Officer (DHO), the overseer of all health services in the district, was contacted for approval to use the facility and their patients, and a letter of support for this research was issued. The nurse/midwife in-charge of the maternity unit was contacted for support. She informed the members of the staff in the unit about the study and urged them to offer daily support to the data collection. We obtained consent from the pregnant women or their mother/husband. If a woman was unable to write, we obtained a fingerprint of her thumb of non-dominant hand as a proof of her consent to voluntarily participate in the research study. Data and blood sample collection only commenced after the women gave voluntary consent to participate in the study.

### 2.3. IFA Pills

The Malawian government uses prenatal supplements as a short-term solution to fight micronutrient malnutrition (particularly iron deficiency anemia) in pregnant women and improve the health status of mothers and newborns. All pregnant women, regardless of hematological status or the trimester of pregnancy, receive prenatal supplements of pills of combined iron and folic acid from the first antenatal care visit to delivery. Each tablet contains 60 mg iron (ferrous) and 0.25 mg folic acid, taken once a day. Every month, the woman gets a new supply without a check if she actually utilized the prescription given the previous month. The program is run on the assumption that women understand the need to take the supplements, despite documented evidence of poor compliance due to side effects and women forgetting to take the pills [18,19]. Monitoring prenatal supplementation intake has been continued because it is the most feasible way to fight micronutrient malnutrition and improve health outcomes at least in the short term, compared to diet, considering the current social economic status of Malawians.

### 2.4. Demographics, Antenatal Care, and Maternal Anthropometrics

Using a short questionnaire, the basic characteristics of the pregnant woman were collected: age of the mother, gestation age, gravidity (number of pregnancies), parity (number of deliveries), education level of the mother, and area of residence.

The questionnaire also contained questions on antenatal care that the pregnant woman was able to access during pregnancy. This included information on IFA supplement use, which was obtained from self-reports and confirmed by checking medical records (health passport book, a little handbook that contains all medical information and previous treatments; the women carry the book everywhere). We used the same questions as those used in the Malawi demographic and health survey [6]. The questions ask if the participant received or bought IFA supplements in pregnancy; if she took any of the IFA pills; how many pills she was able to take during pregnancy. Our questionnaire also included questions on the number of antenatal visits the woman received, at what gestation of the pregnancy (trimester) did she start prenatal care, and reasons for starting prenatal care early or late [19]. Information about vaccinations, malaria prophylaxis, and use of anti-helminthes (anti-worms) during pregnancy was abstracted from the medical record (health passport book). We also obtained medical history about sickness during the current pregnancy, such as anemia (blood transfusions) and if the pregnant woman was on any long-term treatment or chronic diseases such as diabetes, hypertension and asthma/allergies. History of contraceptive use prior to the current pregnancy was obtained [20,21].

Maternal weight was taken on the day of the survey just before delivery of the baby using Seca weighing scale (Seca, Chino, CA, USA). Weight was measured to the nearest 0.1 kg in mothers. The midwives performed the anthropometric measurements on pregnant women who participated in the study. The women’s height was measured using a stadiometer. They had to remove anything that she was wearing on her head and ensure that her braided hair was as flat as possible by loosening her hair and pressing her headpiece. The women also had to remove their shoes and heavy clothing (subjects were dressed in light clothes) during the anthropometric measurement process. Height was recorded to the nearest 0.1 cm. Most of the maternal characteristics are routinely documented on the labor-monitoring chart; therefore, only the parameters that are not available from the chart were asked to avoid duplication.

### 2.5. Blood Specimen Collection

We collected blood samples from women before they gave birth. Upon obtaining consent and explaining the procedure, the women was asked to sit or lie on a bed, as per their preference. A tourniquet was applied around the upper left arm and a 70% alcohol swab was used to clean the skin on the site (cubital). A 10 mL syringe with 21-gauge needle (SkyRun Pharma Co., Ltd., Nanjing, China) was then used to withdraw a venous whole blood sample from the cubital region.

Each blood specimen was collected in two different sample tubes of 5 mL each—one for serum and the other for red blood cell (RBC) folate analysis. The third specimen type was taken using a Microcuvette from the same 10 mL to measure iron status (hemoglobin). The blood sample for folate was allowed to flow freely into the specimen bottle by vacuum pressure, after piercing the rubber cover with the needle of the syringe.

The sample for RBC folate analysis was put in a 5 mL tube containing an anticoagulant (ethylenediaminetetraacetic acid, EDTA). The EDTA ensured that the sample did not clot, and that there was adequate plasma for analysis of hematocrit (needed for calculation of RBC folate later). The tube was then gently shaken to mix the blood and the anticoagulant. The serum folate sample was put in a tube without an anticoagulant to allow cells to separate from the serum after clotting. This tube had a gel to separate the red cells from the serum after the clotting of cells. The samples were kept at room temperature for 2–6 h, until they were transferred to a laboratory outside the Bwaila maternity wing (research site hospital) at the African Bible College Clinic—Center for Medical Diagnostics (CMED). At CMED, the RBC folate blood sample was centrifuged and frozen, kept at −80 °C until transfer by air to South Africa.

The folic acid analysis was done in South Africa by Lancet laboratories (Lancet, Johannesburg, South Africa) using ARCHITECT assay kits (Abbott Ireland, Longford, Ireland) on the ARCHITECT *i* system. The hemoglobin measurements were taken at bedside using a Hemocue Hb 201+ (HemoCue America, Brea, CA, USA).

### 2.6. Statistical Analyses

All data analyses were conducted using SPSS version 24 (SPSS Inc., Armonk, NY, USA) and SAS version 9.4 (SAS Institute, Cary, NC, USA). Descriptive statistics were used to calculate frequencies and mean and standard deviation values of variables. We also determined associations between maternal health and compliance to IFA factors, and hemoglobin and folate status using correlation analyses. Finally, multivariable logistic regression models were used to determine the degree of influence of iron and folic acid supplements on LBW infants as an outcome after controlling for covariates. Covariates were maternal age, education, total number of pregnancies, first prenatal visit trimester, and total number of prenatal visits.

## 3. Results

Table 1 shows that the mothers’ age, residence, gravidity (number of pregnancies), parity, number of prenatal care clinic visits, and trimester of starting intake of IFA pills did not differ between mothers who gave birth to normal weight versus LBW newborns. Factors that differentiated the two groups were trimester of first prenatal clinic visit (*p* = 0.003) and gestation age (*p* < 0.001).

Table 2 shows a comparison of mothers’ variables between normal weight and LBW newborns. Mean hemoglobin status between mothers of normal weight and LBW newborns were 11.4 ± 1.6 g/dL and 9.4 ± 1.6 g/dL (*p* < 0.001), respectively. The difference in serum folate between mothers of normal weight and LBW newborns was not significant—9.1 ± 4.4 nmol/L and 7.5 ± 3.6 nmol/L, respectively (*p* = 0.230). The same was observed with RBC folate status between normal weight and LBW—494.6 ± 413 nmol/L and 489.8 ± 181.1 nmol/L, respectively (*p* = 0.942).

**Figure 1:**
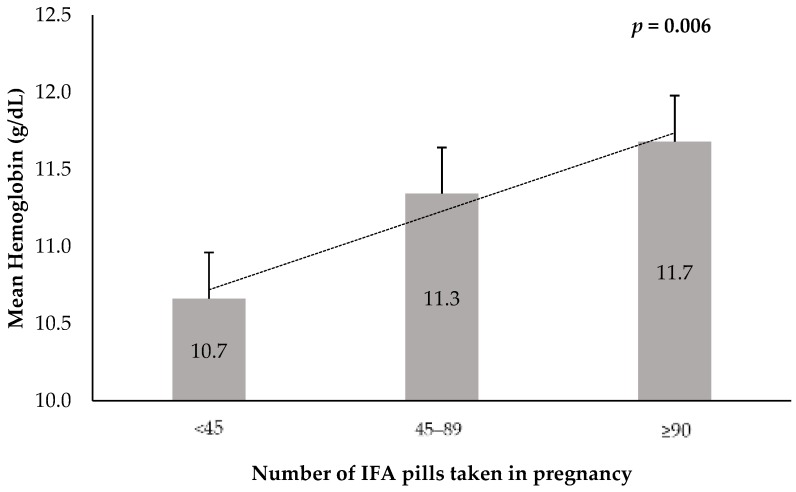
Mean maternal hemoglobin levels before delivery, by number of IFA pills taken during pregnancy.

IFA pills taken by the women during pregnancy and the mean maternal hemoglobin levels before pregnancy are shown in Figure 1. Maternal hemoglobin levels were positively associated with the number of IFA pills taken. Mean hemoglobin levels were 10.7 g/dL, 11.3 g/dL, and 11.7 g/dL in response to the reported number of IFA pills taken from <45, 45–89, to ≥90, respectively (*p* = 0.006).

The correlation of IFA pills taken with mothers’ height, maternal hemoglobin, and serum folate were positive with a Spearman’s correlation coefficient of 0.1759 (*p* = 0.019); 0.1846 (*p* = 0.010); and 0.1839 (*p* = 0.070), respectively (Table 3). The results of multivariable odds ratio (OR) for LBW and IFA pills intake and other variables are shown in Table 4. The OR was not significant for age categories, residence, gravidity, parity, education levels, number of prenatal clinic visits, and trimester of starting IFA pill intake. IFA supplement intake during pregnancy reduced the risk of delivering a LBW newborn. Women who took more than 60 IFA pills had lower risk (OR = 0.15, CI: 0.03, 0.70, *p* = 0.033) compared with the reference group who took ≤30 pills after controlling for maternal age, education, total number of pregnancies, first prenatal visit trimester, and total number of prenatal visits. Risk of LBW was lower for a gestation age ≥37 week (OR = 0.13, CI: 0.05, 0.35, *p* < 0.001) than a gestation age <37 weeks. Women who started prenatal care visit in the second or third trimester had a lower OR of LBW (OR = 0.16, CI: 0.05, 0.51, *p* = 0.002) compared to those who started in the first trimester.

## 4. Discussion

The present study reported that the self-reported intake of IFA reduced the risk of LBW. Women who took more than 60 IFA supplements (pills) had significantly lower odds of delivering LBW babies, compared with pregnant women who took ≤30 pills. The more prenatal IFA pills were taken by pregnant women, the lower the risk of LBW. Similarly, a study by Nisar and colleagues, which used nationally representative data of Pakistan (demographic and health survey), found that self-reported intake of IFA pills of any amount during pregnancy was positively associated with better perceived birth size and birth weight. Any amount of IFA pills taken was associated with a reduced risk (by 18%) of having a smaller-than-average newborn [22]. Another study in India, which also used a national data set to examine the relationship between self-reported intake of IFA pills in pregnancy and LBW risk, found an inverse association. They found that at the population level, in a context where the burden of anemia is severe (prevalence ≥ 40%), IFA pills taken during pregnancy were significantly associated with low LBW. They concluded that the measures to improve the implementation of the prenatal supplementation program would likely help address India’s burden of LBW [23]. The situation in India applies to Malawi because they are both developing countries with existing food security challenges in its communities, have a high prevalence of anemia, and prenatal IFA supplements have proven to be effective.

The compliance to prenatal IFA supplement use among pregnant women was directly associated with increased levels of maternal hemoglobin in the current study. This is in agreement with many other studies that show that maternal hemoglobin has modifying effects on infant birth weight in women receiving prenatal iron-containing supplements [8]. In a randomized double blind multicenter clinical trial (*n* = 18,775 participants) in China, it was found that supplementation of iron and folic acid, folic acid alone, or multiple micronutrient supplements (formulated by the United Nations) impacted birth weight, depending on hemoglobin status of the pregnant woman. Folic acid did not have much impact on birth weight; perhaps, this could explain our results above that although there is a positive association, it was not a statistically significant relationship. The supplement has to have a significant change on maternal hemoglobin levels, and hemoglobin modifies the birth weight of the newborn [8], with more impact seen in those with better hemoglobin status at baseline. Those receiving folic acid supplements alone did not have significant better birth outcomes but those with combined pills or multiple micronutrients did. Another study showed that supplementation did not just improve newborn anthropometric measurements, but also reduced the prevalence of anemia in mothers [12]. Steer also found that low hemoglobin is associated with LBW [10].

We found that pregnant women who self-reported taking supplements consistently (at least two months) lowered their risk of delivering a LBW newborn significantly. After establishing that IFA impacts biomarkers of the hematological status of pregnant women and that biomarkers of supplement use (hemoglobin and folate) have modifying effects on birth weight, we wanted to examine if birth outcomes could be linked to “self-reported” IFA use during pregnancy. If self-reported use of IFA pills during pregnancy is validated to predict birth outcomes in Malawi, it could strengthen the lessons given in prenatal clinics as a part of the anemia prevention program. It could also help reduce the cost of a routine hemoglobin check, which are rarely done due to the lack of resources in Malawi (only 30 out 213 women had their hemoglobin levels tested during prenatal care).

In the present study, pregnant women whose prenatal care began in the second or third trimester had a lower risk of delivering LBW infants, compared to those whose prenatal care began in the first trimester. Most pregnant women in Malawi visit prenatal care clinics in the second trimester. Those who visit in the first trimester are more likely to have medical problems and commence prenatal care immediately after treatment of those issues. Therefore, the cause for LBW in these babies is likely a medical problem, which necessitated early prenatal care. However, there were women without issues in early pregnancy who stayed home until the second trimester and delivered healthier babies because of a healthy pregnancy; this may not necessarily be due to the timing of the first prenatal care visit.

The biggest challenge identified was sustained intake of IFA pills longer than three months. Most pregnant women reported negligence, reduced supply of IFA supplements, and late start of prenatal clinic attendance as major reasons for poor compliance. Now that we have demonstrated that self-reported intake of prenatal supplements is a valid tool to predict hematological status of biomarkers and birth out comes of pregnant women, it is high time that a monitoring mechanism is put in place to evaluate pregnant women’s compliance to IFA every time they visit a prenatal clinic. The monitoring mechanism could combine pill counting [18] to ensure that compliance is being achieved and pregnant women adhere to intake of IFA pills supplements. Pill counting has been employed successfully to improve adherence to ant-retroviral drugs in the treatment of HIV/AIDS [24]. Another method being used in Malawi is directly observed treatment (DOT), where a patient takes drugs under the observation of a medical practitioner or a trusted guardian. DOT is being used in the treatment of tuberculosis (the first two weeks) and malaria prophylaxis in pregnant women at the prenatal clinic. An effective monitoring and evaluation mechanism would make the people of Malawi realize the benefits of the IFA supplementation program and make it more successful in lowering the prevalence of LBW. The education of pregnant women on the benefits of IFA on their newborns would also yield positive results, because negligence was one of the major reasons for poor compliance. Lowering the risk of LBW has long-term benefits, such as reducing infant and child mortality, decreasing stunting prevalence, which in turn has economic returns and national development [25].

Maternal nutrition status is a major determinant of LBW; however, social and demographic factors have also been reported as significant [26,27]. Muula et al. found in 2008 that maternal education was associated with birth weight. Those with low levels of education are more likely to have a LBW children than those with a higher education [26]. It was also found that parity was a factor of LBW, i.e., the first delivery was likely to be LBW, compared to later born children. The Malawi Demographic and Health Survey demonstrates that birth orders as well as the maternal age are factors for LBW. The firstborn child is likely to have low weight than the second, and younger mothers and those giving birth after 35 years of age are more likely to deliver a LBW child [6]. The wealth index of the family had a positive association with birth weight, as well as attainment of secondary education and location of residence (for example, cities vs. rural areas) [27].

The significance of this study is that the validation of the self-reported intake of IFA with biomarkers (Hb and serum and red blood cell folate) evaluated the effectiveness of the program in general in improving the health of women and birth outcomes, and monitoring approaches used by the Malawi government for the largest anemia program in the country. This research provides feedback to government programs and non-profit organizations working in nutrition in Malawi on the efficacy of prenatal supplements to reduce LBW. Overall, the research results provide evidence that the self-reported intake of IFA by pregnant women can be used as a monitoring tool for compliance to prenatal supplements. The study also introduces the innovation of using the placenta, which is normally discarded in Malawi, to determine compliance to and efficacy of prenatal supplements taken during pregnancy and its impact on newborn health.

## 5. Conclusions

In conclusion, self-reported IFA supplement intake in pregnancy is a predictor of birth weight. Compliance to prenatal IFA supplement use can be improved if the Malawian government improves the supply of IFA pills in clinics, ensures that every pregnant woman attends prenatal clinics early in pregnancy, and possibly adds pill count to the questions asked at a follow up before supplying the next batch of IFA pills. Further studies using a more nationally representative sample of pregnant women should be done to determine compliance to IFA and birth outcomes in Malawi.

## Figures and Tables

**Table 1:** Characteristics between mothers of normal weight and low birth weight newborns.

	Birth Weight of Newborn				
	Normal Weight (*n* = 179)		Low Birth Weight (*n* = 34)		
	*N*	%	*N*	%	*p* Value
Age					
<30 y	138	77.1	27	79.4	0.767
≥30 y	41	22.9	7	20.6	
Residence					
Rural	58	32.4	12	35.3	0.722
Peri-Urban	68	38.0	12	35.3	
Urban	53	29.6	10	29.4	
Gravidity					
1	54	30.3	16	47.1	0.058
≥2	124	69.7	18	52.9	
Missing	1				
Parity					
0	55	30.7	15	45.5	0.098
≥1	124	69.3	18	54.6	
Missing	1				
Gestation Weeks					
<37	38	24.1	22	71.0	<0.001
≥37	120	76.0	9	29.0	
Missing	24				
Education Level					
≤Primary	98	54.8	24	70.6	0.087
≥Secondary	81	45.3	10	29.4	
Trimester of First Prenatal Visit					
First	11	6.2	7	21.9	0.003
Second	134	75.3	23	71.9	
Third	33	18.5	2	6.3	
Missing	3				
No. of Prenatal Clinic Visits					
<4	97	55.8	21	63.6	0.401
≥4	77	44.3	12	36.4	
Missing	6				
No. of IFA Pills Taken during Pregnancy					
<45	17	54.8	64	36.6	0.084
45–89	12	38.7	77	44.0	
≥90	2	6.5	34	19.4	
Missing	7				
Trimester IFA Pill Intake Started					
First	13	8.4	7	25.0	0.088
Second	126	81.3	18	64.3	
Third	16	10.3	3	10.7	
Missing	30				

IFA, iron and folic acid; *p*-value by Chi-square test.

**Table 2:** Differences in characteristics between mothers of normal weight and low birth weight newborns.

	Birth Weight						
	Normal Weight (*n* = 179)			Low Birth Weight (*n* = 34)			
	*N*	Mean	SD	*N*	Mean	SD	*p* Value
Mother’s Variables							
Body Weight-1 (kg)	159	60.1	9.9	26	54.6	8.4	0.008
Body Weight-2 (kg)	125	66.4	11.2	16	59.5	8.6	0.019
Height (cm)	154	155.5	5.6	31	151.0	6.4	<0.001
Hb-1 (g/dL)	23	9.7	1.4	6	7.6	1.7	0.005
Hb-2 (g/dL)	172	11.4	1.6	27	9.4	1.6	<0.001
Serum Folate (nmol/L)	87	9.1	4.4	14	7.5	3.6	0.230
RBC Folate (nmol/L)	87	494.6	413.0	14	489.8	181.1	0.942
Placenta Weight (g)	145	591.4	129.2	26	455.6	153.9	<0.001

Body Weight-1: First prenatal visit weight, data were extracted from health passport book; Body Weight-2: Measured prior to delivery; Hb-1: Hemoglobin status at first prenatal clinic visit, data was extracted from health passport book; Hb-2: Hemoglobin level measured prior to delivery; IFA, iron and folic acid.

**Table 3:** Spearman’s correlation of prenatal iron and folic acid (IFA) supplement use with maternal characteristics.

		Body Weight-1	Body Weight-2	Height	Hemo-Globin	Serum Folate	RBC Folate
No. of IFA Pills	Corr. Coeff.	0.0599	0.0042	0.1759	0.1846	0.1839	−0.0329
	*p* value	0.426	0.961	0.019	0.010	0.070	0.748
	*n*	179	136	178	193	98	98
Body Weight-1	Corr. Coeff.		0.8910	0.1794	0.1025	0.0697	0.0266
	*p* value		<0.001	0.022	0.180	0.526	0.809
	*n*		129	164	173	85	85
Body Weight-2	Corr. Coeff.			0.2121	0.1489	0.0537	−0.0287
	*p* value			0.015	0.084	0.654	0.811
	*n*			131	136	72	72
Height	Corr. Coeff.				0.1819	−0.1529	−0.0432
	*p* value				0.016	0.141	0.679
	*n*				174	94	94
Hemoglobin	Corr. Coeff.					0.3905	−0.0167
	*p* value					<0.001	0.869
	*n*					100	100
Serum Folate	Corr. Coeff.						0.2502
	*p* value						0.012
	*n*						101

Body Weight-1: First prenatal visit weight, data were extracted from health passport book; Body Weight-2: Measured prior to delivery; Hemoglobin, serum and RBC folate samples were taken prior to delivery.

**Table 4:** Multivariable odds ratios (OR) and 95% confidence intervals (CIs) of low birth weight by its risk factors.

	Low Birth Weight			
	OR *	95% CI		*p* Value
Age (*n* = 204)				
<30 y	1.00			
≥30 y	1.55	0.53	4.56	0.427
Residence (*n* = 204)				
Rural	1.00			
Peri-Urban	1.14	0.44	2.93	0.673
Urban	0.90	0.32	2.54	0.717
Gravidity (*n* = 204)				
1	1.00			
≥2	0.45	0.18	1.14	0.093
Parity (*n* = 204)				
0	1.00			
≥1	0.49	0.19	1.26	0.14
Gestation Weeks (*n* = 181)				
<37	1.00			
≥37	0.13	0.05	0.35	<0.001
Education (*n* = 204)				
≤Primary	1.00			
≥Secondary	0.67	0.29	1.55	0.346
First ANC Visit Trimester (*n* = 204)				
First	1.00			
Second/Third	0.16	0.05	0.51	0.002
No. of Prenatal Clinic Visits (*n* = 204)				
<4	1.00			
≥4	0.51	0.21	1.23	0.13
Trimester IFA Supplements Started (*n* = 159)				
First	1.00			
Second/Third	0.41	0.11	1.63	0.208
No. of IFA Pills Taken in Pregnancy (*n* = 199)				
≤30	1.00			
31–60	0.57	0.23	1.41	0.447
>60	0.15	0.03	0.70	0.033
No. of IFA Pills Taken in Pregnancy (*n* = 199)				
<45	1.00			
45–89	0.54	0.22	1.32	0.842
≥90	0.24	0.05	1.14	0.145

ANC, antenatal care; IFA, iron and folic acid. * Adjusted for maternal age, education, total number of pregnancies, first prenatal visit trimester, and total number of prenatal visits.

## Abbreviations

LBW	low birth weight
IFA	iron and folic acid
Hb	Hemoglobin
RBC	red blood cell
OR	odds ratios
